# Tiered restrictions for COVID-19 in England: knowledge, motivation and self-reported behaviour

**DOI:** 10.1016/j.puhe.2021.12.016

**Published:** 2022-03

**Authors:** L.E. Smith, H.W.W. Potts, R. Amlȏt, N.T. Fear, S. Michie, G.J. Rubin

**Affiliations:** a King's College London, Institute of Psychiatry, Psychology and Neuroscience, UK; b NIHR Health Protection Research Unit in Emergency Preparedness and Response, UK; c University College London, Institute of Health Informatics, UK; d UK Health Security Agency, Behavioural Science and Insights Unit, UK; e King's Centre for Military Health Research and Academic Department of Military Mental Health, UK; f University College London, Centre for Behaviour Change, UK

**Keywords:** COVID-19, Adherence, Restrictions, Guidance, Physical distancing, Social distancing

## Abstract

**Objectives:**

To test whether public knowledge and confidence in one's understanding of the local restrictions, motivation to adhere to local restrictions, and self-reported behaviour (going out for exercise, to work, socially) differed according to tier level.

**Study design:**

Cross-sectional, nationally representative, online survey of 1728 participants living in England (data collection: 26 to 28 October 2020).

**Methods:**

We conducted logistic regression analyses to investigate whether knowledge of restrictions, confidence in knowledge of restrictions, motivation to adhere to restrictions, and self-reported behaviour were associated with personal characteristics and tier.

**Results:**

Between 81% (tier 2) and 89% (tier 3) of participants correctly identified which tier they lived in. Knowledge of specific restrictions was variable. 73% were confident that they understood which tier was in place in their local area, whereas 71% were confident they understood the guidance in their local area. Confidence was associated with being older and living in a less deprived area. 73% were motivated to adhere to restrictions in their local area. Motivation was associated with being female and older. People living in tiers with greater restrictions were less likely to report going out to meet people from another household socially; reported rates of going out for exercise and for work did not differ.

**Conclusions:**

Although recognition of local tier level was high, knowledge of specific guidance for tiers was variable. There was some indication that nuanced guidance (e.g. behaviour allowed in some settings but not others) was more poorly understood than guidance which was absolute (i.e. behaviour is either allowed or not allowed).

## Introduction

The first COVID-19 pandemic restrictions in England were nationwide. As the pandemic progressed, different infection rates in areas across the country led to a more localised approach being applied. For example, the city of Leicester continued to follow more stringent restrictions when those in the rest of the country were eased on 4 July 2020.^1^^,^^2^ Over time, additional restrictions were imposed and eased in other areas.^3^ This led to a complicated patchwork of restrictions throughout England. On 14 October 2020, a three tiered system was introduced in an attempt to simplify local restrictions for COVID-19.^4^ English areas were assigned to tiers by the UK Government based on transmission levels, rates of increase of infection, age distributions, and the capacity of local healthcare services. The main restrictions that were in place in each tier are shown in Table 1. In response to growing infection rates, a second period of national lockdown was imposed from 5 November to 2 December 2020,^5^ before reverting to a slightly stricter three-tier system.^6^ The devolved nations have each taken their own approach, with Scotland implementing a five-level system,^7^ and Wales implementing a four-level alert system.^8^ Northern Ireland also implemented localised COVID-19 restrictions.^9^

**Table 1:** Main restrictions in place in each tier from October to November 2020 in England.

Tier 1	Tier 2	Tier 3
Up to six people could meet indoors, outdoors in private gardens, and outdoors in public spaces	Up to six people could meet outdoors in private gardens and outdoors in public spaces	Up to six people could meet outdoors in public spaces
	No household mixing indoors	No household mixing indoors
Hospitality venues remained open, but the majority had to close between 10pm and 5am	Hospitality venues remained open, but the majority had to close between 10pm and 5am	Hospitality venues such as pubs and bars had to close, unless they operated as a restaurant, serving ‘substantial meals’. Closures between 10pm and 5am remained in place
		Travelling to areas in other tiers discouraged

Knowledge of the restrictions in place to prevent the spread of COVID-19 has been sub-optimal throughout the pandemic.^10^ People have found guidance about social distancing and self-isolation confusing,^11^ with frequent changes to the guidance contributing to this.^12^^,^^13^ Throughout the pandemic, the use of clear and specific guidance has been emphasised to promote adherence to restrictions.^14^ As clarity in the guidance around tiers appears to increase as restrictions tighten, it is plausible that understanding of the guidance and potentially motivation to adhere,^15^ is higher in tiers with more stringent restrictions.

Regional restrictions have also been used in other countries, for example, color-coded zones (e.g. red, orange, yellow zones) have been used in Italy, France, and the Quebec province in Canada, and tiered local alert levels have been used in New Zealand.^16^ At the time of writing, tiers are still being used in Scotland. Although the influence of tiered restrictions on infection rates has been investigated,^17^, ^18^, ^19^ there is limited information available on how well members of the public understand and tiered levels of restrictions and how restrictions affect general behaviour.

The aim of this study was to investigate people's knowledge of and confidence in understanding restrictions in place in their local area, motivation to adhere to these restrictions, and self-reported behaviour (going out for exercise, to work, and socially) under the tier system implemented in October 2020, and whether there were differences by tier.

## Methods

### Design

Throughout the COVID-19 pandemic, BMG Research (a Market Research Society Company Partner) has been conducting a series of cross-sectional nationally representative surveys for the Department of Health and Social Care, England. We analyzed these data as part of the COVID-19 Rapid Survey of Adherence to Interventions and Responses (CORSAIR) study. For this paper, we used data collected on 26 to 28 October 2020 (wave 31) as it gave insight into participants’ knowledge and self-reported behaviour while the tier system was in place in October to November 2020. As participants were asked to report their behaviour in the previous 7 days, this was the only survey wave that reported solely on the time under the English tier system. Additional methodological details are described in Smith et al., 2021.^20^

### Participants

Participants were eligible for the survey if they were aged 16 years or over and living in the United Kingdom. Of 2043 participants who completed wave 31, 1728 lived in England. Participants were recruited from two specialist research panel providers (Respondi, n = 50,000; Savanta, n = 31,500). These are panels of people who have signed up to take part in online surveys. Panel members who are eligible to take part in a specific survey based on their characteristics are sent a survey link. In line with industry standards, completion of the survey implies consent to take part. Where a participant's responses do not meet quality assurance standards (e.g. completing the survey too quickly, or giving the same answer for multiple consecutive questions), that participant's data are removed from the sample. Quota sampling (based on age and gender combined) was used to ensure that the sample was broadly representative of the population. Targets for recruitment were set based on participants' age and gender. Where quotas were already filled, participants of that age and gender combination were not able to complete the survey. Participants were reimbursed for having completed the survey in points, which could be redeemed as cash, gift vouchers or charitable donations (up to 70p per survey).

### Measures

We asked participants which COVID-19 ‘local alert level’ they thought applied to where they lived. Response options were ‘Tier 1 (medium)’, ‘Tier 2 (high)’, ‘Tier 3 (very high)’, and ‘don't know’. We recoded participants as knowing their COVID-19 level if they correctly identified which tier their local area was in. For this variable, we coded answers of ‘don't know’ as incorrect. We used a 4-point scale to measure participants' confidence in their understanding of the tier that applied to where they lived (recoded to a binary variable: ‘not at all confident’ and ‘not very confident’ vs ‘fairly confident’ and ‘very confident’).

To investigate knowledge of individual guidance in one's local area, we asked participants a series of statements about the guidance on socializing ‘where [you] live’. These statements covered meeting in groups outdoors in public spaces, in private gardens and indoors; meeting with members of your ‘support’ or ‘childcare’ bubbles^a^ outdoors and indoors; staying overnight in someone else's home; travelling to other parts of the United Kingdom for leisure; sharing a car with someone not in your household; and taking part in group worship. Possible answers were ‘true’, ‘false’, and ‘don't know’. We also asked participants how confident they were that they understood the guidance currently in place in their local area (recoded to a binary variable using groupings described above).

Participants were asked how motivated they were to adhere to restrictions put in place by the Government in their local area on a 4-point scale (recoded to a binary variable: ‘not at all’ and ‘slightly’ vs ‘quite a bit’ and ‘strongly’).

We asked participants how many times in the last seven days they had left their home for different reasons, including for exercise; spending time outdoors for recreational purposes; work; and meeting up with friends and/or family that they did not live with. We grouped going out for a walk or some exercise and spending time outdoors for recreational purposes into a single variable. We recoded these variables to indicate whether participants reported going out for that reason at least once in the last week.

#### Personal characteristics

Participants were asked their age, gender, whether there was a dependent child in the household, their employment status, socio-economic grade, highest educational or professional qualification, ethnicity, and how many people lived in their household.

Participants had to provide their full postcode, from which they were assigned region, index of multiple deprivation and whether they were categorised as living in a tier 1, 2, or 3 area at the time of data collection.

### Ethics

This work was conducted as a service evaluation of the Department of Health and Social Care's public communications campaign, rather than being constituted as research, and, following advice from King's College London Research Ethics Subcommittee, was exempt from ethical approval.

### Analysis

Logistic regression analyses were undertaken to investigate whether personal characteristics and tier level were associated with: knowledge of which tier you live in, confidence in understanding your local tier, confidence in guidance in place in your local area, motivation to adhere to restrictions in your local area, and self-reported outings (separate analyses for outings for exercise or recreation outside, going to work, and meeting up with friends or family from another household). We restricted analyses of people going out to work to those who reported working (n = 868). For each set of analyses, we ran univariable analyses and multivariable analyses (controlling for region, gender, age [raw and quadratic], presence of dependent children in the household, employment status, socio-economic grade, index of multiple deprivation, highest educational or professional qualification, ethnicity, and living alone). We controlled for these variables based on theoretical grounds and the results of previous analyses on this data set.^20^, ^21^, ^22^

We assumed that people might be most likely to understand a rule if it directly related to activities that they personally engaged in. We, therefore, conducted an additional analysis, restricting the sample only to those who reported having met up with friends and family not living in their household in the last week. We created a single binary variable denoting if participants knew the guidance in their local area regarding meeting in groups in public spaces, in private gardens, and indoors. For this variable, we coded answers of ‘don't know’ as incorrect. We used logistic regressions to investigate associations between knowledge about meeting others and personal characteristics and tier. In other words, we tested whether people who met up with others knew the guidance about meeting up with others.

To take account of the number of analyses undertaken (n = 15), we only report narratively on adjusted results that remained statistically significant after a Bonferroni correction (*P* < .003). Uncorrected *P*-values are given in the results tables.

## Results

### Knowledge and confidence in understanding the tier system

There were no observable differences between tier levels in terms of correct identification of which tier level applied. Overall, between 81.1% (tier 2) and 89.1% (tier 3) of people knew their tier level (see Supplementary materials for full breakdown). When adjusting for other personal characteristics, correct knowledge of which tier applied was associated with being female and older (see Table 2).

**Table 2:** Associations between correct knowledge of tier, and participant characteristics and tier. Bolding signifies significant results (*P* < 0.003).

		Incorrect knowledge of tier or did not know n = 282, n (%)	Correct knowledge of tier n = 1446, n (%)	Odds ratio for correct knowledge of tier (95% CI)	*P*-value	Adjusted odds ratio for correct knowledge of tier (95% CI)^a^	*P*-value
Region	Overall	–	–	χ^2^(2) = 7.4	0.03	χ^2^(2) = 5.8	0.06
	Midlands (East and West)	58 (16.1)	303 (83.9)	Reference	–	Reference	–
	North England (Northeast, Northwest, Yorkshire, and the Humber)	62 (12.7)	425 (87.3)	1.31 (0.89–1.93)	0.17	1.32 (0.87–2.02)	0.20
	South England (Southeast, Southwest, London, and East of England)	162 (18.4)	718 (81.6)	0.85 (0.61–1.18)	0.33	0.86 (0.60–1.24)	0.42
Gender	Male	145 (19.4)	604 (80.6)	Reference	–	Reference	–
	Female	136 (14.0)	834 (86.0)	1.47 (1.14–1.90)	0.003	**1.68 (1.27 to 2.22)**	**<0.001**
Age	Raw age (range 16–90)	M = 40.9, SD = 17.8	M = 50.7, SD = 17.4	**1.03 (1.02 to 1.04)**	**<0.001**	**1.02 (1.01 to 1.03)**	**<0.001**
Age: quadratic (age-mean)^2^	**-**	–	–	–	–	0.9995 (0.9990–1.0000)	0.05
Presence of dependent children in the household	None	158 (13.2)	1037 (86.8)	Reference	–	Reference	–
	Child present	124 (23.3)	409 (76.7)	**0.50 (0.39 to 0.65)**	**<0.001**	0.65 (0.47–0.89)	0.01
Employment status	Not working	118 (14.2)	715 (85.8)	Reference	–	Reference	–
	Working	153 (17.6)	715 (82.4)	0.77 (0.59–1.00)	0.05	0.96 (0.70–1.32)	0.82
Socio-economic grade	ABC1	188 (15.6)	1016 (84.4)	Reference	–	Reference	–
	C2DE	87 (18.0)	395 (82.0)	0.84 (0.64–1.11)	0.22	1.07 (0.78–1.45)	0.69
Index of multiple deprivation	1st quartile (least deprived) to 4th quartile (most deprived)	M = 2.8, SD = 1.1	M = 2.6, SD = 1.1	**0.81 (0.72 to 0.91)**	**<0.001**	0.90 (0.79–1.02)	0.09
Highest educational or professional qualification	GCSE/vocational/A-level/no formal qualifications	202 (16.5)	1022 (83.5)	Reference	–	Reference	–
	Degree or higher (Bachelor's, Master's, PhD)	80 (15.9)	424 (84.1)	1.05 (0.79–1.39)	0.75	1.30 (0.94–1.79)	0.11
Ethnicity	Overall	–	–	**χ**^**2**^**(2)=43.5**	**<0.001**	χ2(2) = 7.3	0.03
	White British	193 (13.5)	1237 (86.5)	Reference	–	Reference	–
	White Other	34 (29.6)	81 (70.4)	**0.37 (0.24 to 0.57)**	**<0.001**	0.55 (0.34–0.89)	0.02
	Black/Asian/Mixed/Other	52 (29.5)	124 (70.5)	**0.37 (0.26 to 0.53)**	**<0.001**	0.69 (0.45–1.05)	0.08
Living alone	Not living alone	228 (16.3)	1170 (83.7)	Reference	–	Reference	–
	Living alone	54 (16.4)	276 (83.6)	1.00 (0.72–1.38)	0.98	0.66 (0.46–0.97)	0.03
Tier (local COVID-19 alert level)	Overall	–	–	χ^2^(2) = 8.3	0.02	χ^2^(2) = 3.9	0.14
	Tier 1 (medium)	133 (16.0)	700 (84.0)	Reference	–	Reference	–
	Tier 2 (high)	122 (18.9)	525 (81.1)	0.82 (0.62–1.07)	0.14	0.97 (0.70–1.34)	0.86
	Tier 3 (very high)	27 (10.9)	221 (89.1)	1.56 (1.00–2.42)	0.05	1.77 (0.93–3.37)	0.08

a Adjusted for region, gender, age (raw and quadratic), presence of dependent children in the household, employment status, socio-economic grade, index of multiple deprivation, highest educational or professional qualification, ethnicity, and living alone.

72.8% (95% CI 70.7%–74.9%) of respondents reported being confident that they understood which tier applied to their local area. Confidence was associated with being older and living in a less deprived area; there was no association with tier (see Supplementary materials).

### Knowledge and confidence in understanding local guidance

Knowledge of local guidance was mixed (see Table 3). Incorrect knowledge was particularly common for guidance about: staying overnight in someone else's home, travelling to other parts of the United Kingdom for leisure, sharing a car with someone not in your household, and taking part in group worship.

**Table 3:** Knowledge of guidance in your local area. Bolding denotes the correct answer.

You can…	Tier 1 (medium), total n = 833, n (%)			Tier 2 (high), total n = 647, n (%)			Tier 3 (very high), total n = 248, n (%)		
	True	False	Don't know	True	False	Don't know	True	False	Don't know
Meet in groups of up to six people from different households outdoors, in private gardens	**610 (73.2)**	133 (16.0)	90 (10.8)	**340 (52.6)**	230 (35.5)	77 (11.9)	47 (19.0)	**184 (74.2)**	17 (6.9)
Meet in groups of up to six people from different households outdoors, in a public space, for example, a park	**675 (81.0)**	91 (10.9)	67 (8.0)	**433 (66.9)**	145 (22.4)	69 (10.7)	**130 (52.4)**	93 (37.5)	25 (10.1)
Meet in groups of up to six people from different household indoors, for example, in a pub, restaurant or café or at someone's home	**549 (65.9)**	184 (22.1)	100 (12.0)	139 (21.5)	**438 (67.7)**	70 (10.8)	39 (15.7)	**189 (76.2)**	20 (8.1)
Meet with your support or childcare bubble indoors, if you have one	**594 (71.3)**	80 (9.6)	159 (19.1)	**431 (66.6)**	117 (18.1)	99 (15.3)	**146 (58.9)**	64 (25.8)	38 (15.3)
Meet with your support or childcare bubble outdoors, if you have one	**625 (75.0)**	57 (6.8)	151 (18.1)	**450 (69.6)**	94 (14.5)	103 (15.9)	**162 (65.3)**	46 (18.5)	40 (16.1)
Stay overnight in someone else's home	**276 (33.1)**	369 (44.3)	188 (22.6)	69 (9.1)	**508 (78.5)**	80 (12.4)	25 (10.1)	**197 (79.4)**	26 (10.5)
Travel to other parts of the United Kingdom for leisure (e.g. for a day trip or to see friends or family)	**415 (49.8)**	237 (28.5)	181 (21.7)	**188 (29.1)**	330 (51.0)	129 (19.9)	37 (14.9)	**190 (76.6)**	21 (8.5)
Share a car with someone not in your household but are advised to take precautions like wearing a mask or opening the windows	**494 (59.3)**	174 (20.9)	165 (19.8)	**209 (32.3)**	287 (44.4)	151 (23.3)	**82 (33.1**)	127 (51.2)	39 (15.7)
Take part in group worship at a place of worship	**365 (43.8)**	171 (20.5)	297 (35.7)	**213 (32.9)**	231 (35.7)	203 (31.4)	67 (27.0)	**112 (45.2)**	69 (27.8)

70.9% (95% CI 68.7%–73.0%) of respondents were confident that they understood the guidance currently in place in their local area. Confidence was associated with being older and living in a less deprived area. There was no association with tier (see Supplementary materials).

#### Meeting up with people from another household

There were 602 respondents (34.8%) who reported having met up with friends or family they did not live with, in the last week. Among these respondents, 50.8% (95% CI 46.8%–54.8%) knew the guidance surrounding meeting up with people from another household in their local area. Knowledge differed by tier, with people in tier 1 being most likely to know the guidance (see Table 4). Correct knowledge of the guidance was also associated with living in less deprived areas.

**Table 4:** Associations between correct knowledge of guidance about meeting others from another household, and participant characteristics and tier. Bolding signifies significant results (*P* < 0.003).

		Incorrect knowledge of guidance n = 296, n (%)	Correct knowledge of guidance n = 306, n (%)	Odds ratio for correct knowledge of guidance (95% CI)	*P*-value	Adjusted odds ratio for correct knowledge of guidance (95% CI)^a^	*P*-value
Region	Overall	–	–	χ^2^(2) = 10.4	0.01	χ^2^(2) = 10.3	0.01
	Midlands (East and West)	59 (48.0)	64 (52.0)	Reference	–	Reference	–
	North England (Northeast, Northwest, Yorkshire, and the Humber)	79 (61.7)	49 (38.3)	0.57 (0.35–0.94)	0.03	0.56 (0.33–0.97)	0.04
	South England (Southeast, Southwest, London, and East of England)	158 (45.0)	193 (55.0)	1.13 (0.75–1.70)	0.57	1.16 (0.74–1.82)	0.52
Gender	Male	135 (54.9)	111 (45.1)	Reference	–	Reference	–
	Female	159 (45.0)	194 (55.0)	1.48 (1.07–2.06)	0.02	1.46 (1.02–2.08)	0.04
Age	Raw age (range 16–90)	M = 43.8, SD = 19.6	M = 46.0, SD = 17.5	1.01 (1.00–1.02)	0.15	1.00 (0.99–1.01)	0.78
Age: quadratic (age-mean)^2^	**-**	–	–	–	–	**0.9988 (0.9982 to 0.9994)**	**<0.001**
Presence of dependent children in the household	None	184 (47.9)	200 (52.1)	Reference	–	Reference	–
	Child present	112 (51.4)	106 (48.6)	0.87 (0.62–1.21)	0.41	0.75 (0.49–1.13)	0.17
Employment status	Not working	131 (49.1)	136 (50.9)	Reference	–	Reference	–
	Working	161 (49.2)	166 (50.8)	0.99 (0.72–1.37)	0.97	0.79 (0.53–1.19)	0.26
Socio-economic grade	ABC1	194 (48.0)	210 (52.0)	Reference	–	Reference	–
	C2DE	92 (51.1)	88 (48.9)	0.88 (0.62–1.26)	0.49	0.95 (0.65–1.40)	0.79
Index of multiple deprivation	1st quartile (least deprived) to 4th quartile (most deprived)	M = 2.7, SD = 1.1	M = 2.3, SD = 1.1	**0.71 (0.62 to 0.83)**	**<0.001**	**0.75 (0.64 to 0.88)**	**0.001**
Highest educational or professional qualification	GCSE/vocational/A-level/no formal qualifications	210 (51.3)	199 (48.7)	Reference	–	Reference	–
	Degree or higher (Bachelor's, Master's, PhD)	86 (44.6)	107 (55.4)	1.31 (0.93–1.85)	0.12	1.35 (0.91–2.00)	0.14
Ethnicity	Overall	–	–	χ^2^(2) = 7.7	0.02	χ^2^(2) = 4.2	0.12
	White British	232 (47.3)	259 (52.7)	Reference	–	Reference	–
	White Other	29 (47.5)	32 (52.5)	0.99 (0.58–1.68)	0.97	1.15 (0.62–2.11)	0.66
	Black/Asian/Mixed/Other	33 (68.8)	15 (31.3)	0.41 (0.22–0.77)	0.01	0.50 (0.25–1.02)	0.06
Living alone	Not living alone	226 (48.2)	243 (51.8)	Reference	–	Reference	–
	Living alone	70 (52.6)	63 (47.4)	0.84 (0.57–1.23)	0.37	0.78 (0.49–1.24)	0.29
Tier (local COVID-19 alert level)	Overall	–	–	**χ**^**2**^**(2)=47.8**	**<0.001**	**χ**2**(2)=28.0**	**<0.001**
	Tier 1 (medium)	124 (36.6)	215 (63.4)	Reference	–	Reference	–
	Tier 2 (high)	141 (65.9)	73 (34.1)	**0.30 (0.21 to 0.43)**	**<0.001**	**0.32 (0.21 to 0.49)**	**<0.001**
	Tier 3 (very high)	31 (63.3)	18 (36.7)	**0.33 (0.18 to 0.62)**	**0.001**	0.41 (0.18–0.94)	0.04

a Adjusted for region, gender, age (raw and quadratic), presence of dependent children in the household, employment status, socio-economic grade, index of multiple deprivation, highest educational or professional qualification, ethnicity, and living alone.

### Motivation to adhere to restrictions and self-reported behaviour

73.1% (95% CI 71.1%–75.2%) of respondents were motivated to adhere to restrictions in place in their local area. Motivation to adhere to restrictions in place in one's local area was associated with being female and older; there was no association with tier (see Supplementary materials).

The percentage of people who reported having gone out in the last week to meet friends or family that they did not live with was lower in tiers 2 and 3 compared with tier 1 (see Table 5). Self-reported outings for exercise or recreation and going out to work did not differ by tier.

**Table 5:** Self-reported outings in the last 7 days, by tier. Bolding signifies significant results (*P* < 0.003).

Tier (local COVID-19 alert level)	Been out for a walk or some other exercise or to spend time outdoors for recreational purposes (including to sit in parks etc.)					
	Did not go out in last week n = 551, n (%)	Went out in last week n = 1177, n (%)	Odds ratio for having been out at least once in the last week (95% CI)	*P*-value	Adjusted odds ratio for having been out at least once in the last week (95% CI)^a^	*P*-value
Overall	–	–	χ^2^(2) = 7.2	0.03	χ^2^(2) = 6.3	0.04
Tier 1 (medium)	247 (29.7)	586 (70.3)	Reference	–	Reference	–
Tier 2 (high)	208 (32.1)	439 (67.9)	0.89 (0.71–1.11)	0.30	0.89 (0.69–1.15)	0.38
Tier 3 (very high)	96 (38.7)	152 (61.3)	0.67 (0.50–0.90)	0.01	0.57 (0.36–0.88)	0.01

	Been out to work (in those who reported working)					
	Did not go out in last week n = 363, n (%)	Went out in last week n = 505, n (%)	Odds ratio for having been out at least once in the last week (95% CI)	*P*-value	Adjusted odds ratio for having been out at least once in the last week (95% CI)^b^	*P*-value
Overall	–	–	χ^2^(2) = 0.2	0.89	χ^2^(2) = 4.1	0.13
Tier 1 (medium)	161 (41.5)	227 (58.5)	Reference	–	Reference	–
Tier 2 (high)	143 (41.4)	202 (58.6)	1.00 (0.75–1.34)	0.99	0.89 (0.63–1.26)	0.52
Tier 3 (very high)	59 (43.7)	76 (56.3)	0.91 (0.62–1.36)	0.65	0.52 (0.28–0.98)	0.04

	Been out to meet up with friends and/or family that you do not live with					
	Did not go out in last week n = 1126, n (%)	Went out in last week n = 602, n (%)	Odds ratio for having been out at least once in the last week (95% CI)	*P*-value	Adjusted odds ratio for having been out at least once in the last week (95% CI)^a^	*P*-value

Overall	–	–	**χ**^**2**^**(2)=36.9**	**<0.001**	**χ**2**(2)=12.8**	**0.002**
Tier 1 (medium)	494 (59.3)	339 (40.7)	Reference	–	Reference	–
Tier 2 (high)	433 (66.9)	214 (33.1)	0.72 (0.58–0.89)	0.003	0.74 (0.58–0.96)	0.02
Tier 3 (very high)	199 (80.2)	49 (19.8)	**0.36 (0.25 to 0.51)**	**<0.001**	**0.44 (0.27 to 0.70)**	**0.001**

a Adjusted for region, gender, age (raw and quadratic), presence of dependent children in the household, employment status, socio-economic grade, index of multiple deprivation, highest educational or professional qualification, ethnicity, and living alone.

b Adjusted for region, gender, age (raw and quadratic), presence of dependent children in the household, socio-economic grade, index of multiple deprivation, highest educational or professional qualification, ethnicity, and living alone.

Going out for a walk or recreation was associated with region (with those in South England being more likely to than those in the Midlands), living in a less deprived area, and identifying as White Other (compared with White British; see Supplementary materials). Going out to work was associated with lower socio-economic grade (C2DE compared with ABC1; see Supplementary materials). Meeting up with others from another household was associated with region (although no individual region reached our threshold for statistical significance), younger age, living in a less deprived area, and living alone (see Supplementary materials). People identifying as Black, Asian, Mixed, or Other ethnicities were less likely to meet others from another household (compared with White British).

## Discussion

Our analysis indicates that, in the case of the English tier system, recognition of which tier applied to a person's local area was high (81%–89%), but knowledge of the specific restrictions that were in place was poorer (29%–81%). Women and older participants were more likely to correctly identify their local tier. This is in line with other research finding that, overall, women and older adults have better knowledge, and confidence in their knowledge, about COVID-19.^20^^,^^23^^,^^24^ This may be due to higher health literacy in these groups.^25^

Clearly, people do not need to understand all the rules that apply to their local area. There is no reason, for example, for people without children to have detailed knowledge of the rules relating to childcare. However, even restricting our analyses to the most common activity that is governed by COVID-19 restrictions (meeting up with people from another household), we found that people who reported that they had met with friends or family in the last week had poor knowledge about the restrictions for meeting people. Only 50% correctly identified the specific restrictions that applied in their local area. Guidance was particularly poorly understood by people living in tier 2 and in more deprived areas. In part, this may relate to the various nuances that existed within this guidance (e.g. specifying how many people could meet, and where meetings could occur). Restrictions that are absolute (e.g. behaviour is or is not permitted) may be clearer and more easily understood.

Although knowledge of specific guidance was poor, people's confidence in their understanding of guidance was higher. This may reflect the gap between actual and perceived knowledge that is seen in other health-related situations.^26^^,^^27^ Although we did not find an association between motivation to adhere to local restrictions and living in a more deprived area, poorer confidence in knowledge about the tier system and local restrictions was associated with living in a more deprived area and younger age. Poorer adherence in these groups has been a common theme throughout the pandemic.^20^ Greater attention to ensuring that regulations and guidelines are clearly communicated may be helpful to improve adherence in these groups.

We found a complex and varied impact of tiered guidance on general behaviour. All behaviours we investigated (going out for a walk or exercise, to work, and to meet friends or family from another household) were allowed in all tiers. Going out to meet someone from another household was associated with tier level, with fewer people reporting meeting up with others in higher tiers. One explanation for this is that people had control over this behaviour, which they adjusted in accordance with higher perceptions of risk in their local area. Another explanation is that people had less opportunity to meet up, for example in indoor settings such as restaurants. There was no evidence that going out to work differed by tier. However, going out for a walk or exercise, a behaviour which participants had control over, but which was not explicitly mentioned by tiered guidance, showed a trend towards declining in higher tiers, suggesting a spill-over effect of the guidance that may have related to risk perception.^28^ COVID-19 restrictions have increased sedentary behaviour.^29^^,^^30^ Going out for a walk or exercise alone or with members of one's own household is a low risk activity with respect to COVID-19 transmission and should be encouraged for its effects on well-being. There is also evidence that those who are consistently inactive are at a higher risk of hospitalization, admission to intensive care, and death from COVID-19.^31^ It is, therefore, a concern if people avoid these behaviours.

Strengths of this study include that data were collected soon after the behaviour, limiting recall bias. However, behaviour was self-reported and may have been subject to social desirability bias. The use of an anonymous online survey should have mitigated the impact of this. Limitations include the use of cross-sectional data meaning that we cannot infer causation. While the sample was recruited to be representative of the population based on age and gender, we cannot be certain that the views and behaviours of survey respondents are representative of those of the general population. We did not investigate whether participants who reported meeting up with people from other households did so in a manner adherent to the restrictions in their local area.

Results from our study suggest that although overall tier level was well recognised, individual restrictions were poorly understood. Clear, unambiguous restrictions (e.g. behaviour is or is not allowed), where possible, are likely to be better understood than nuanced restrictions. Better communications may be needed to reach people in groups with poorer understanding and confidence in their understanding. It was notable that two behaviours over which people had control (meeting others and going out for exercise) declined with more restrictive tier. This suggests that the impact of tiers is not solely due to the specific guidance involved, but has a broader impact.

## Author statements

### Ethical approval

This work was conducted as a service evaluation of the Department of Health and Social Care's public communications campaign and, following advice from King's College London Research Ethics Subcommittee, was exempt from ethical approval.

### Funding

This work was funded by the 10.13039/501100000272National Institute for Health Research (NIHR) Health Services and Delivery Research programme. LS, RA, and GJR are supported by the 10.13039/100018336National Institute for Health Research Health Protection Research Unit (NIHR HPRU) in Emergency Preparedness and Response, a partnership between the UK Health Security Agency, King's College London and the University of East Anglia. RA is also supported by the 10.13039/100018336NIHR HPRU in Behavioural Science and Evaluation, a partnership between the UK Health Security Agency and the University of Bristol. The views expressed are those of the authors and not necessarily those of the NIHR, the UK Health Security Agency, the Department of Health and Social Care or the Ministry of Defence. Surveys were commissioned and funded by 10.13039/501100000276Department of Health and Social Care (DHSC), with the authors providing advice on the question design and selection. DHSC had no role in analysis, decision to publish, or preparation of the manuscript. Preliminary results were made available to DHSC and the UK's Scientific Advisory Group for Emergencies.

### Competing interests

All authors had financial support from 10.13039/501100000272NIHR for the submitted work. RA is an employee of the UK Health Security Agency; HWWP has received additional salary support from Public Health England and NHS England; HWWP receives consultancy fees to his employer from Ipsos MORI and has a PhD student who works at and has fees paid by Astra Zeneca; NTF is a participant of an independent group advising NHS Digital on the release of patient data. All authors are participants of the UK's Scientific Advisory Group for Emergencies or its subgroups. There are no other financial relationships with any organizations that might have an interest in the submitted work in the previous three years and no other relationships or activities that could appear to have influenced the submitted work.

## Data availability statement

The data are owned by England's Department of Health and Social Care, so no additional data are available from the authors.

## Footnotes

a Support and childcare bubbles were introduced to provide social support to people who live alone and to allow informal childcare for people who have children. Guidance about support bubbles can be found here: https://www.gov.uk/guidance/making-a-support-bubble-with-another-household. Guidance about childcare bubbles can be found here: https://www.gov.uk/guidance/making-a-childcare-bubble-with-another-household.
