# Corrigendum to ‘Tiered restrictions for COVID-19 in England: Knowledge, motivation and self-reported behaviour’ [Public Health 204 (2022) 33–39]

**DOI:** 10.1016/j.puhe.2022.05.014

**Published:** 2022-08

**Authors:** L.E. Smith, H.W.W. Potts, R. Amlȏt, N.T. Fear, S. Michie, G.J. Rubin

**Affiliations:** a King's College London, Institute of Psychiatry, Psychology and Neuroscience, UK; b NIHR Health Protection Research Unit in Emergency Preparedness and Response, UK; c University College London, Institute of Health Informatics, UK; d UK Health Security Agency, Behavioural Science and Insights Unit, UK; e King's Centre for Military Health Research and Academic Department of Military Mental Health, UK; f University College London, Centre for Behaviour Change, UK

The authors regret that there are some errors with this manuscript.^1^

There is an issue with labelling for socio-economic grade. The item for this variable asks participants to state the profession of the highest earner in the household. We categorised participants into two groups: highest earner works in a manual occupation, and highest earner does not work in a manual occupation. The levels of these variables are referred to in some of our project's manuscripts as “socio-economic grade C1DE” and “socio-economic grade ABC1” respectively (see Table 1). These would be better denoted as “highest earner in household works in a manual occupation” and “highest earner in household does not work in a manual occupation”. This labelling error came about through multiple iterations of documents.

**Table 1:** Socio-economic grade variable labelling.

**Which of the following best describes the occupation of the member of your household with the largest income (the chief income earner)? Please select one answer** Please indicate to which occupational group the Chief Income Earner in your household belongs, or which group fits best. The Chief Income Earner is the person in your household with the largest income. If the Chief Income Earner is retired and has an occupational pension please answer for their most recent occupation. If the Chief Income Earner is not in paid employment but has been out of work for less than 6 months, please answer for their most recent occupation	Labelling as given in manuscript	Amended labelling for manuscript	Socio-economic grade (corrected)
**Semi or unskilled manual work** (*e.g.* Manual workers, all apprentices to skilled trades, Caretaker, Park keeper, non-HGV driver, shop assistant)	C2DE	Highest earner in household works in a manual occupation	C2DE
**Skilled manual worker***(e.g. Skilled Bricklayer, Carpenter, Plumber, Painter, Bus/Ambulance Driver, HGV driver, AA patrolman, pub/bar worker, etc.)*	C2DE	Highest earner in household works in a manual occupation	C2DE
**Supervisory or clerical/junior managerial/professional/administrative***(e.g. Office worker, Student Doctor, Foreman with 25*+ *employees, salesperson, etc.)*	ABC1	Highest earner in household does not work in a manual occupation	ABC1
**Intermediate managerial/professional/administrative***(e.g. Newly qualified (under 3 years) doctor, Solicitor, Board director small organisation, middle manager in large organisation, principle officer in civil service/local government)*	ABC1	Highest earner in household does not work in a manual occupation	ABC1
**Higher managerial/professional/administrative***(e.g. Established doctor, Solicitor, Board Director in a large organisation (200+ employees, top level civil servant/public service employee)*	ABC1	Highest earner in household does not work in a manual occupation	ABC1
**Student**	ABC1	Highest earner in household does not work in a manual occupation	ABC1
**Casual worker – not in permanent employment**	ABC1	Highest earner in household does not work in a manual occupation	C2DE
**Housewife/Homemaker**	ABC1	Highest earner in household does not work in a manual occupation	C2DE
**Retired and living on state pension**	ABC1	Highest earner in household does not work in a manual occupation	C2DE
**Unemployed or not working due to long-term sickness**	ABC1	Highest earner in household does not work in a manual occupation	C2DE
**Full-time carer of another household member**	ABC1	Highest earner in household does not work in a manual occupation	C2DE
Other	Missing	Missing	Missing

For this study, 482 participants (27.9%) reported that the highest earner in their household worked in a manual occupation (highest earner did not work in a manual occupation: n = 1204, 69.7%; missing data [reported “other” and so could not be categorised]: n = 42, 2.4%). When using socio-economic grade, 764 participants (44.2%) were categorised as belonging to the C2DE group (ABC1: n = 922, 53.4%; missing data: n = 42, 2.4%).

A sensitivity analysis adjusting for socio-economic grade rather than the highest earner being a manual worker indicated that there were no meaningful differences in other analyses investigating the test, trace, and isolate system, and no difference to the conclusions drawn.^2^

The authors would like to apologise for any inconvenience caused.

## Funding statement

This work was funded by the National Institute for Health Research (10.13039/100006662NIHR) Health Services and Delivery Research programme (NIHR project reference number (11/46/21)). Surveys were commissioned and funded by Department of Health and Social Care (DHSC), with the authors providing advice on the question design and selection. LS, RA and GJR are supported by the National Institute for Health Research Health Protection Research Unit (10.13039/100018336NIHR HPRU) in Emergency Preparedness and Response, a partnership between the UK Health Security Agency, King's College London and the University of East Anglia. RA is also supported by the NIHR HPRU in Behavioural Science and Evaluation, a partnership between the UK Health Security Agency and the University of Bristol. HWWP has received funding from Public Health England and NHS England. NTF is part funded by a grant from the UK Ministry of Defence. The views expressed are those of the authors and not necessarily those of the NIHR, UK Health Security Agency, the Department of Health and Social Care or the Ministry of Defence. The Department of Health and Social Care funded data collection (no grant number).

## Competing interests

All authors had financial support from 10.13039/501100000272NIHR for the submitted work. RA is an employee of the UK Health Security Agency; HWWP received additional salary support from Public Health England and NHS England; HWWP receives consultancy fees to his employer from Ipsos MORI and has a PhD student who works at and has fees paid by Astra Zeneca. At the time of writing GJR is acting as an expert witness in an unrelated case involving 10.13039/100016309Bayer PLC, supported by LS. NTF is a participant of an independent group advising NHS Digital on the release of patient data. All authors were participants of the UK's Scientific Advisory Group for Emergencies or its subgroups.
