# Supplementary material for: Tiered restrictions for COVID-19 in England: knowledge, motivation and self-reported behaviour
Source: Public Health. 2022 Mar;204:33–9. doi: 10.1016/j.puhe.2021.12.016 (PMC8820758; doi:10.1016/j.puhe.2021.12.016)
Supplement: Multimedia component 1 [file mmc1.docx]

# **Supplementary materials**

Table 1. Self-reported COVID-19 local alert level, by tier.

|  | | Which of the three Covid local alert levels applies to where you live? | | | |
| --- | --- | --- | --- | --- | --- |
|  |  | Tier 1 (medium) | Tier 2 (high) | Tier 3 (very high) | Don’t know |
| Alert level at time | Medium (tier 1) | 700 (84.0) | 72 (8.6) | 10 (1.2) | 51 (6.1) |
|  | High (tier 2) | 40 (6.2) | 525 (81.1) | 44 (6.8) | 38 (5.9) |
|  | Very high (tier 3) | 6 (2.4) | 14 (5.6) | 221 (89.1) | 7 (2.8) |

Table 2. Associations between confidence in understanding of local tier, and participant characteristics and tier level. Bolding signifies significant results (*p*<.003).

|  |  | Not at all confident / not very confident / don’t know n=470, n (%) | Fairly confident / very confident n=1258, n (%) | Odds ratio for confidence in understanding of tier (95% CI) | p-value | Adjusted odds ratio for confidence in understanding of tier (95% CI)† | p-value |
| --- | --- | --- | --- | --- | --- | --- | --- |
| Region | Overall | - | - | χ^2^(2)=0.2 | .90 | χ^2^(2)=0.1 | .97 |
|  | England – Midlands (East and West) | 96 (26.6) | 265 (73.4) | Reference | - | Reference | - |
|  | North England (North East, North West, Yorkshire and the Humber) | 136 (27.9) | 351 (72.1) | 0.93 (0.69 to 1.27) | .67 | 1 (0.72 to 1.39) | .99 |
|  | South England (South East, South West, London, East of England) | 238 (27.0) | 642 (73.0) | 0.98 (0.74 to 1.29) | .87 | 0.97 (0.72 to 1.3) | .85 |
| Gender | Male | 188 (25.1) | 561 (74.9) | Reference | - | Reference | - |
|  | Female | 280 (28.9) | 690 (71.1) | 0.83 (0.67 to 1.02) | .08 | 0.86 (0.69 to 1.08) | .20 |
| Age | Raw age (range 16 to 90) | M=44.1, SD=16.8 | M=51.0, SD=17.9 | **1.022 (1.016 to 1.029)** | **<.001** | **1.02 (1.01 to 1.03)** | **<.001** |
| Age: quadratic (age-mean)^2^ | **-** | - | - | - | - | 1.0005 (1.0001 to 1.0009) | .02 |
| Presence of dependent children in the household | None | 297 (24.9) | 898 (75.1) | Reference | - | Reference | - |
|  | Child present | 173 (32.5) | 360 (67.5) | **0.69 (0.55 to 0.86)** | **.001** | 1.01 (0.77 to 1.32) | .94 |
| Employment status | Not working | 205 (24.6) | 628 (75.4) | Reference | - | Reference | - |
|  | Working | 254 (29.3) | 614 (70.7) | 0.79 (0.64 to 0.98) | .03 | 1.00 (0.78 to 1.29) | .99 |
| Socio-economic grade | ABC1 | 328 (27.2) | 876 (72.8) | Reference | - | Reference | - |
|  | C2DE | 130 (27.0) | 352 (73.0) | 1.01 (0.80 to 1.29) | .91 | 1.22 (0.95 to 1.58) | .12 |
| Index of multiple deprivation | 1^st^ quartile (least deprived) to 4^th^ quartile (most deprived) | M=2.8, SD=1.1 | M=2.5, SD=1.1 | **0.78 (0.71 to 0.86)** | **<.001** | **0.82 (0.74 to 0.91)** | **<.001** |
| Highest educational or professional qualification | GCSE/vocational/A-level/No formal qualifications | 349 (28.5) | 875 (71.5) | Reference | - | Reference | - |
|  | Degree or higher (Bachelor’s, Master’s, PhD) | 121 (24.0) | 383 (76.0) | 1.26 (0.99 to 1.60) | .06 | 1.43 (1.10 to 1.87) | .01 |
| Ethnicity | Overall | - | - | χ^2^(2)=4.8 | .09 | χ^2^(2)=0.6 | .97 |
|  | White British | 373 (26.1) | 1057 (73.9) | Reference | - | Reference | - |
|  | White Other | 36 (31.3) | 79 (68.7) | 0.77 (0.51 to 1.17) | .22 | 0.96 (0.61 to 1.52) | .87 |
|  | Black/Asian/Mixed/Other | 58 (33.0) | 118 (67.0) | 0.72 (0.51 to 1) | .05 | 0.96 (0.66 to 1.39) | .83 |
| Living alone | Not living alone | 386 (27.6) | 1012 (72.4) | Reference | - | Reference | - |
|  | Living alone | 84 (25.5) | 246 (74.5) | 1.12 (0.85 to 1.47) | .43 | 0.88 (0.65 to 1.20) | .42 |
| Tier (local COVID-19 alert level) | Overall | - | - | χ^2^(2)=7.9 | .02 | χ^2^(2)=5.8 | .05 |
|  | Tier 1 (medium) | 209 (25.1) | 624 (74.9) | Reference | - | Reference | - |
|  | Tier 2 (high) | 201 (31.1) | 446 (68.9) | 0.74 (0.59 to 0.93) | .01 | 0.92 (0.70 to 1.20) | .52 |
|  | Tier 3 (very high) | 60 (24.2) | 188 (75.8) | 1.05 (0.75 to 1.46) | .77 | 1.55 (0.97 to 2.49) | .07 |

† Adjusted for region, gender, age (raw and quadratic), presence of dependent children in the household, employment status, socio-economic grade, index of multiple deprivation, highest educational or professional qualification, ethnicity, and living alone.

Table 3. Associations between confidence in understanding of local guidance, and participant characteristics and tier. Bolding signifies significant results (*p*<.003).

|  |  | Not at all confident / not very confident / don’t know n=503, n (%) | Fairly confident / very confident n=1225, n (%) | Odds ratio for confidence in understanding of local guidance (95% CI) | p-value | Adjusted odds ratio for confidence in understanding of local guidance (95% CI)† | p-value |
| --- | --- | --- | --- | --- | --- | --- | --- |
| Region | Overall | - | - | χ^2^(2)=2.9 | .24 | χ^2^(2)=2.2 | .33 |
|  | England – Midlands (East and West) | 106 (29.4) | 255 (70.6) | Reference | - | Reference | - |
|  | North England (North East, North West, Yorkshire and the Humber) | 155 (31.8) | 332 (68.2) | 0.89 (0.66 to 1.20) | .44 | 0.98 (0.71 to 1.33) | .88 |
|  | South England (South East, South West, London, East of England) | 242 (27.5) | 638 (72.5) | 1.10 (0.84 to 1.44) | .51 | 1.17 (0.88 to 1.55) | .29 |
| Gender | Male | 216 (28.8) | 533 (71.2) | Reference | - | Reference | - |
|  | Female | 285 (29.4) | 685 (70.6) | 0.97 (0.79 to 1.20) | .81 | 1.00 (0.80 to 1.25) | 1.00 |
| Age | Raw age (range 16 to 90) | M=45.1, SD=16.9 | M=50.8, SD=18.0 | **1.018 (1.012 to 1.024)** | **<.001** | **1.02 (1.01 to 1.03)** | **<.001** |
| Age: quadratic (age-mean)^2^ | **-** | - | - | - | - | 1.0006 (1.0002 to 1.001) | .004 |
| Presence of dependent children in the household | None | 328 (27.4) | 867 (72.6) | Reference | - | Reference | - |
|  | Child present | 175 (32.8) | 358 (67.2) | 0.77 (0.62 to 0.97) | .02 | 1.07 (0.82 to 1.39) | .60 |
| Employment status | Not working | 224 (26.9) | 609 (73.1) | Reference | - | Reference | - |
|  | Working | 269 (31.0) | 599 (69.0) | 0.82 (0.66 to 1.01) | .06 | 1.05 (0.82 to 1.34) | .71 |
| Socio-economic grade | ABC1 | 351 (29.2) | 853 (70.8) | Reference | - | Reference | - |
|  | C2DE | 141 (29.3) | 341 (70.7) | 1.00 (0.79 to 1.26) | .97 | 1.17 (0.91 to 1.50) | .22 |
| Index of multiple deprivation | 1^st^ quartile (least deprived) to 4^th^ quartile (most deprived) | M=2.8, SD=1.1 | M=2.5, SD=1.1 | **0.80 (0.73 to 0.88)** | **<.001** | 0.86 (0.77 to 0.95) | .003 |
| Highest educational or professional qualification | GCSE/vocational/A-level/No formal qualifications | 369 (30.1) | 855 (69.9) | Reference | - | Reference | - |
|  | Degree or higher (Bachelor’s, Master’s, PhD) | 134 (26.6) | 370 (73.4) | 1.19 (0.94 to 1.50) | .14 | 1.38 (1.07 to 1.79) | .01 |
| Ethnicity | Overall | - | - | χ^2^(2)=7.5 | .02 | χ^2^(2)=2.1 | .34 |
|  | White British | 396 (27.7) | 1034 (72.3) | Reference | - | Reference | - |
|  | White Other | 41 (35.7) | 74 (64.3) | 0.69 (0.46 to 1.03) | .07 | 0.78 (0.50 to 1.22) | .28 |
|  | Black/Asian/Mixed/Other | 63 (35.8) | 113 (64.2) | 0.69 (0.49 to 0.95) | .03 | 0.80 (0.56 to 1.16) | .24 |
| Living alone | Not living alone | 409 (29.3) | 989 (70.7) | Reference | - | Reference | - |
|  | Living alone | 94 (28.5) | 236 (71.5) | 1.04 (0.80 to 1.35) | .78 | 0.87 (0.65 to 1.17) | .35 |
| Tier (local COVID-19 alert level) | Overall | - | - | χ^2^(2)=11.1 | .004 | χ^2^(2)=2.6 | .27 |
|  | Tier 1 (medium) | 211 (25.3) | 622 (74.7) | Reference | - | Reference | - |
|  | Tier 2 (high) | 211 (32.6) | 436 (67.4) | **0.70 (0.56 to 0.88)** | **.002** | 0.81 (0.62 to 1.05) | .11 |
|  | Tier 3 (very high) | 81 (32.7) | 167 (67.3) | 0.70 (0.51 to 0.95) | .02 | 0.80 (0.51 to 1.26) | .34 |

† Adjusted for region, gender, age (raw and quadratic), presence of dependent children in the household, employment status, socio-economic grade, index of multiple deprivation, highest educational or professional qualification, ethnicity, and living alone.

Table 4. Associations between motivation to adhere to restrictions in place in your local area, and participant characteristics and tier. Bolding signifies significant results (*p*<.003).

|  |  | Not at all / slightly n=464, n (%) | Quite a bit / strongly n=1264, n (%) | Odds ratio for being motivated (95% CI) | p-value | Adjusted odds ratio for being motivated (95% CI)† | p-value |
| --- | --- | --- | --- | --- | --- | --- | --- |
| Region | Overall | - | - | χ^2^(2)=4.1 | .13 | χ^2^(2)=2.1 | .34 |
|  | England – Midlands (East and West) | 82 (22.7) | 279 (77.3) | Reference | - | Reference | - |
|  | North England (North East, North West, Yorkshire and the Humber) | 133 (27.3) | 354 (72.7) | 0.78 (0.57 to 1.07) | .13 | 0.85 (0.6 to 1.2) | .36 |
|  | South England (South East, South West, London, East of England) | 249 (28.3) | 631 (71.7) | 0.74 (0.56 to 0.99) | .04 | 0.79 (0.58 to 1.08) | .14 |
| Gender | Male | 222 (29.6) | 527 (70.4) | Reference | - | Reference | - |
|  | Female | 240 (24.7) | 730 (75.3) | 1.28 (1.03 to 1.59) | .02 | **1.51 (1.19 to 1.92)** | **.001** |
| Age | Raw age (range 16 to 90) | M=39.9, SD=15.8 | M=52.5, SD=17.3 | **1.044 (1.037 to 1.051)** | **<.001** | **1.04 (1.03 to 1.05)** | **<.001** |
| Age – quadratic (age-mean)^2^ | **-** | - | - | - | - | 1.0001 (0.9996 to 1.0005) | .81 |
| Presence of dependent children in the household | None | 266 (22.3) | 929 (77.7) | Reference | - | Reference | - |
|  | Child present | 198 (37.1) | 335 (62.9) | **0.48 (0.39 to 0.61)** | **<.001** | 0.84 (0.64 to 1.1) | .21 |
| Employment status | Not working | 162 (19.4) | 671 (80.6) | Reference | - | Reference | - |
|  | Working | 292 (33.6) | 576 (66.4) | **0.48 (0.38 to 0.59)** | **<.001** | 0.72 (0.55 to 0.94) | .01 |
| Socio-economic grade | ABC1 | 302 (25.1) | 902 (74.9) | Reference | - | Reference | - |
|  | C2DE | 152 (31.5) | 330 (68.5) | 0.73 (0.58 to 0.92) | .01 | 0.95 (0.73 to 1.23) | .68 |
| Index of multiple deprivation | 1^st^ quartile (least deprived) to 4^th^ quartile (most deprived) | M=2.8, SD=1.1 | M=2.5, SD=1.1 | **0.81 (0.74 to 0.89)** | **<.001** | 0.87 (0.78 to 0.97) | .01 |
| Highest educational or professional qualification | GCSE/vocational/A-level/No formal qualifications | 311 (25.4) | 913 (74.6) | Reference | - | Reference | - |
|  | Degree or higher (Bachelors, Masters, PhD) | 153 (30.4) | 351 (69.6) | 0.78 (0.62 to 0.98) | .04 | 1.01 (0.77 to 1.31) | .97 |
| Ethnicity | Overall | - | - | **χ^2^(2)=19.7** | **<.001** | χ^2^(2)=0.1 | .95 |
|  | White British | 353 (24.7) | 1077 (75.3) | Reference | - | Reference | - |
|  | White Other | 43 (37.4) | 72 (62.6) | 0.55 (0.37 to 0.82) | .003 | 1.07 (0.68 to 1.68) | .78 |
|  | Black/Asian/mixed/other | 66 (37.5) | 110 (62.5) | **0.55 (0.39 to 0.76)** | **<.001** | 1.05 (0.72 to 1.53) | .82 |
| Living alone | Not living alone | 393 (28.1) | 1005 (71.9) | Reference | - | Reference | - |
|  | Living alone | 71 (21.5) | 259 (78.5) | 1.43 (1.07 to 1.90) | .02 | 0.96 (0.68 to 1.34) | .80 |
| Tier (local COVID-19 alert level) | Overall | - | - | χ^2^(2)=8.1 | .02 | χ^2^(2)=1.0 | .62 |
|  | Tier 1 (medium) | 198 (23.8) | 635 (76.2) | Reference | - | Reference | - |
|  | Tier 2 (high) | 196 (30.3) | 451 (69.7) | 0.72 (0.57 to 0.90) | .005 | 0.87 (0.66 to 1.15) | .33 |
|  | Tier 3 (very high) | 70 (28.2) | 178 (71.8) | 0.79 (0.58 to 1.09) | .15 | 0.87 (0.53 to 1.42) | .57 |

† Adjusted for region, gender, age (raw and quadratic), presence of dependent children in the household, employment status, socio-economic grade, index of multiple deprivation, highest educational or professional qualification, ethnicity, and living alone.

Table 5. Associations between having been out for exercise or recreation, and participant characteristics. Bolding signifies significant results (*p*<.003).

|  |  | Did not go out in last week n=551, n (%) | Went out in last week n=1177, n (%) | Odds ratio for going out for a walk or recreation (95% CI) | p-value | Adjusted odds ratio for going out for a walk or recreation (95% CI)† | p-value |
| --- | --- | --- | --- | --- | --- | --- | --- |
| Region | Overall | - | - | **χ^2^(2)=16.0** | **<.001** | χ^2^(2)=11.9 | .003 |
|  | England – Midlands (East and West) | 141 (39.1) | 220 (60.9) | Reference | - | Reference | - |
|  | North England (North East, North West, Yorkshire and the Humber) | 165 (33.9) | 322 (66.1) | 1.25 (0.94 to 1.66) | .12 | 1.32 (0.98 to 1.78) | .07 |
|  | South England (South East, South West, London, East of England) | 245 (27.8) | 635 (72.2) | **1.66 (1.28 to 2.15)** | **<.001** | **1.61 (1.22 to 2.10)** | **.001** |
| Gender | Male | 237 (31.6) | 512 (68.4) | Reference | - | Reference | - |
|  | Female | 311 (32.1) | 659 (67.9) | 0.98 (0.80 to 1.20) | .85 | 0.95 (0.76 to 1.17) | .62 |
| Age | Raw age (range 16 to 90) | M=51.3, SD=17.7 | M=48.1, SD=17.8 | **0.990 (0.984 to 0.995)** | **<.001** | 0.994 (0.986 to 1.001) | .09 |
| Age: quadratic (age-mean)^2^ | **-** | - | - | - | - | 1 (0.9996 to 1.0004) | .94 |
| Presence of dependent children in the household | None | 401 (33.6) | 794 (66.4) | Reference | - | Reference | - |
|  | Child present | 150 (28.1) | 383 (71.9) | 1.29 (1.03 to 1.61) | .03 | 1.11 (0.84 to 1.45) | .47 |
| Employment status | Not working | 283 (34.0) | 550 (66.0) | Reference | - | Reference | - |
|  | Working | 260 (30.0) | 608 (70.0) | 1.20 (0.98 to 1.48) | .08 | 1.07 (0.83 to 1.36) | .62 |
| Socio-economic grade | ABC1 | 380 (31.6) | 824 (68.4) | Reference | - | Reference | - |
|  | C2DE | 156 (32.4) | 326 (67.6) | 0.96 (0.77 to 1.21) | .75 | 0.92 (0.72 to 1.17) | .48 |
| Index of multiple deprivation | 1^st^ quartile (least deprived) to 4^th^ quartile (most deprived) | M=2.7, SD=1.1 | M=2.5, SD=1.1 | **0.86 (0.79 to 0.94)** | **.001** | **0.86 (0.77 to 0.94)** | **.002** |
| Highest educational or professional qualification | GCSE/vocational/A-level/No formal qualifications | 411 (33.6) | 813 (66.4) | Reference | - | Reference | - |
|  | Degree or higher (Bachelor’s, Master’s, PhD) | 140 (27.8) | 364 (72.2) | 1.31 (1.05 to 1.65) | .02 | 1.15 (0.89 to 1.48) | .28 |
| Ethnicity | Overall | - | - | **χ^2^(2)=18.0** | **<.001** | **χ^2^(2)=15.8** | **<.001** |
|  | White British | 471 (32.9) | 959 (67.1) | Reference | - | Reference | - |
|  | White Other | 15 (13.0) | 100 (87.0) | **3.27 (1.88 to 5.70)** | **<.001** | **3.29 (1.75 to 6.17)** | **<.001** |
|  | Black/Asian/Mixed/Other | 60 (34.1) | 116 (65.9) | 0.95 (0.68 to 1.32) | .76 | 0.84 (0.58 to 1.21) | .35 |
| Living alone | Not living alone | 423 (30.3) | 975 (69.7) | Reference | - | Reference | - |
|  | Living alone | 128 (38.8) | 202 (61.2) | 0.68 (0.53 to 0.88) | .003 | 0.75 (0.57 to 0.99) | .04 |

† Adjusted for region, gender, age (raw and quadratic), presence of dependent children in the household, employment status, socio-economic grade, index of multiple deprivation, highest educational or professional qualification, ethnicity, and living alone.

Table 6. Associations between having been out to work, and participant characteristics in those who reported working. Bolding signifies significant results (*p*<.003).

|  |  | Did not go out in last week n=363, n (%) | Went out in last week n=505, n (%) | Odds ratio for going out to work (95% CI) | p-value | Adjusted odds ratio for going out to work (95% CI)† | p-value |
| --- | --- | --- | --- | --- | --- | --- | --- |
| Region | Overall | - | - | χ^2^(2)=1.1 | .59 | χ^2^(2)=0.6 | .74 |
|  | England – Midlands (East and West) | 76 (41.8) | 106 (58.2) | Reference | - | Reference | - |
|  | North England (North East, North West, Yorkshire and the Humber) | 92 (39.1) | 143 (60.9) | 1.11 (0.75 to 1.65) | .59 | 0.93 (0.61 to 1.42) | .74 |
|  | South England (South East, South West, London, East of England) | 195 (43.2) | 256 (56.8) | 0.94 (0.66 to 1.33) | .73 | 0.87 (0.60 to 1.26) | .45 |
| Gender | Male | 161 (41.8) | 224 (58.2) | Reference | - | Reference | - |
|  | Female | 201 (42.0) | 278 (58.0) | 0.99 (0.76 to 1.30) | .97 | 1.01 (0.76 to 1.34) | .96 |
| Age | Raw age (range 16 to 90) | M=45.1, SD=14.1 | M=42.2, SD=13.5 | **0.98 (0.97 to 0.99)** | **.002** | 0.98 (0.97 to 1.00) | .03 |
| Age: quadratic (age-mean)^2^ | **-** | - | - | - | - | 1 (0.999 to 1.001) | .59 |
| Presence of dependent children in the household | None | 230 (45.4) | 277 (54.6) | Reference | - | Reference | - |
|  | Child present | 133 (36.8) | 228 (63.2) | 1.42 (1.08 to 1.88) | .01 | 1.18 (0.85 to 1.63) | .33 |
| Socio-economic grade | ABC1 | 274 (48.8) | 287 (51.2) | Reference | - | Reference | - |
|  | C2DE | 82 (28.4) | 207 (71.6) | **2.41 (1.78 to 3.27)** | **<.001** | **2.05 (1.49 to 2.83)** | **<.001** |
| Index of multiple deprivation | 1^st^ quartile (least deprived) to 4^th^ quartile (most deprived) | M=2.4, SD=1.1 | M=2.7, SD=1.1 | **1.27 (1.13 to 1.44)** | **<.001** | 1.18 (1.03 to 1.34) | .02 |
| Highest educational or professional qualification | GCSE/vocational/A-level/No formal qualifications | 214 (39.5) | 328 (60.5) | Reference | - | Reference | - |
|  | Degree or higher (Bachelor’s, Master’s, PhD) | 149 (45.7) | 177 (54.3) | 0.78 (0.59 to 1.02) | .07 | 0.83 (0.61 to 1.14) | .25 |
| Ethnicity | Overall | - | - | χ^2^(2)=1.5 | .48 | χ^2^(2)=0.5 | .76 |
|  | White British | 292 (42.3) | 399 (57.7) | Reference | - | Reference | - |
|  | White Other | 24 (34.8) | 45 (65.2) | 1.37 (0.82 to 2.30) | .23 | 1.15 (0.66 to 2.03) | .62 |
|  | Black/Asian/Mixed/Other | 44 (42.3) | 60 (57.7) | 1.00 (0.66 to 1.51) | .99 | 0.90 (0.57 to 1.43) | .66 |
| Living alone | Not living alone | 298 (40.9) | 430 (59.1) | Reference | - | Reference | - |
|  | Living alone | 65 (46.4) | 75 (53.6) | 0.80 (0.56 to 1.15) | .23 | 0.86 (0.57 to 1.29) | .47 |

† Adjusted for region, gender, age (raw and quadratic), presence of dependent children in the household, socio-economic grade, index of multiple deprivation, highest educational or professional qualification, ethnicity, and living alone.

Table 7. Associations between meeting up with others from another household and participant characteristics. Bolding signifies significant results (*p*<.003).

|  |  | Did not go out in last week n=1126, n (%) | Went out in last week n=602, n (%) | Odds ratio for going out to meet others (95% CI) | p-value | Adjusted odds ratio for going out to meet others (95% CI)† | p-value |
| --- | --- | --- | --- | --- | --- | --- | --- |
| Region | Overall | - | - | **χ^2^(2)=25.4** | **<.001** | **χ^2^(2)=16.2** | **<.001** |
|  | England – Midlands (East and West) | 238 (65.9) | 123 (34.1) | Reference | - | Reference | - |
|  | North England (North East, North West, Yorkshire and the Humber) | 359 (73.7) | 128 (26.3) | 0.69 (0.51 to 0.93) | .01 | 0.69 (0.50 to 0.95) | .02 |
|  | South England (South East, South West, London, East of England) | 529 (60.1) | 351 (39.9) | 1.28 (0.99 to 1.66) | .06 | 1.18 (0.89 to 1.55) | .24 |
| Gender | Male | 503 (67.2) | 246 (32.8) | Reference | - | Reference | - |
|  | Female | 617 (63.6) | 353 (36.4) | 1.17 (0.96 to 1.43) | .13 | 1.1 (0.88 to 1.36) | .40 |
| Age | Raw age (range 16 to 90) | M=51.4, SD=17.0 | M=44.9, SD=18.6 | **0.979 (0.974 to 0.985)** | **<.001** | **0.977 (0.969 to 0.984)** | **<.001** |
| Age: quadratic (age-mean)^2^ | **-** | - | - | - | - | **1.0008 (1.0004 to 1.0011)** | **<.001** |
| Presence of dependent children in the household | None | 811 (67.9) | 384 (32.1) | Reference | - | Reference | - |
|  | Child present | 315 (59.1) | 218 (40.9) | **1.46 (1.18 to 1.81)** | **<.001** | 1.31 (1.01 to 1.70) | .04 |
| Employment status | Not working | 566 (67.9) | 267 (32.1) | Reference | - | Reference | - |
|  | Working | 541 (62.3) | 327 (37.7) | 1.28 (1.05 to 1.57) | .02 | 1.1 (0.86 to 1.41) | .44 |
| Socio-economic grade | ABC1 | 800 (66.4) | 404 (33.6) | Reference | - | Reference | - |
|  | C2DE | 302 (62.7) | 180 (37.3) | 1.18 (0.95 to 1.47) | .14 | 1.15 (0.90 to 1.47) | .25 |
| Index of multiple deprivation | 1^st^ quartile (least deprived) to 4^th^ quartile (most deprived) | M=2.7, SD=1.1 | M=2.5, SD=1.1 | 0.88 (0.80 to 0.96) | .003 | **0.84 (0.76 to 0.93)** | **.001** |
| Highest educational or professional qualification | GCSE/vocational/A-level/No formal qualifications | 815 (66.6) | 409 (33.4) | Reference | - | Reference | - |
|  | Degree or higher (Bachelor’s, Master’s, PhD) | 311 (61.7) | 193 (38.3) | 1.24 (1.00 to 1.53) | .05 | 1.07 (0.84 to 1.37) | .56 |
| Ethnicity | Overall | - | - | **χ^2^(2)=20.6** | **<.001** | **χ^2^(2)=21.2** | **<.001** |
|  | White British | 939 (65.7) | 491 (34.3) | Reference | - | Reference | - |
|  | White Other | 54 (47.0) | 61 (53.0) | **2.16 (1.47 to 3.17)** | **<.001** | 1.39 (0.90 to 2.14) | .14 |
|  | Black/Asian/Mixed/Other | 128 (72.7) | 48 (27.3) | 0.72 (0.51 to 1.02) | .06 | **0.44 (0.30 to 0.66)** | **<.001** |
| Living alone | Not living alone | 929 (66.5) | 469 (33.5) | Reference | - | Reference | - |
|  | Living alone | 197 (59.7) | 133 (40.3) | 1.34 (1.05 to 1.71) | .02 | **1.83 (1.38 to 2.43)** | **<.001** |

† Adjusted for region, gender, age (raw and quadratic), presence of dependent children in the household, employment status, socio-economic grade, index of multiple deprivation, highest educational or professional qualification, ethnicity, and living alone.
